# Supplementary material for: A Sequential Cross-Sectional Analysis Producing Robust Weekly COVID-19 Rates for South East Asian Countries
Source: Viruses. 2023 Jul 18;15(7):1572. doi: 10.3390/v15071572 (PMC10385022; doi:10.3390/v15071572)
Supplement: Supplementary file 1 [file viruses-15-01572-s001.zip › viruses-2503822-supplementary.pdf]

A sequential cross-sectional analysis producing robust weekly Covid-19 rates for South East Asian countries

Amani Almohaimeed and Jochen Einbeck

2023-07-13

Load required packages

```
library(lubridate)
```

```
## Warning: package 'lubridate' was built under R version 4.3.1

##
## Attaching package: 'lubridate'

## The following objects are masked from 'package:base':
##
##     date, intersect, setdiff, union
```

```
library(npmlreg)
```

Reading in data (from local file or dynamically)

```
#covid.data <- read.csv("owid-covid-data.csv")
covid.data<- read.csv("https://covid.ourworldindata.org/data/owid-covid-data.csv")
```

Create data set with weekly raw case and death counts

```
# to create a variable for weeks that starts on Mondays
covid.data$yearweek <-  format(ymd(covid.data$date),"%Y,%W")

# aggregate daily to weekly counts
covid.data2<- aggregate(cbind(new_cases,new_deaths)~location+yearweek, FUN=sum, data=covid.data)

# create a variable with population sizes
covid.pops<- covid.data[match(covid.data2$location, covid.data$location),c("location", "population")]

# merge and label the columns
covid.week.data<-cbind(covid.data2, covid.pops$population)
```

```r
colnames(covid.week.data)<- c(colnames(covid.data2), "population")

# display the first six rows
head(covid.week.data)
```

```
##         location yearweek new_cases new_deaths population
## 1    Afghanistan  2020,00         0          0   41128772
## 2         Africa  2020,00         0          0 1426736614
## 3        Albania  2020,00         0          0    2842318
## 4        Algeria  2020,00         0          0   44903228
## 5 American Samoa  2020,00         0          0      44295
## 6        Andorra  2020,00         0          0      79843
```

Required function to carry out analysis

```r
weekly.covid.rates <- function(week, lag=0,
                               deaths=TRUE,
                               digits=c(4,7),
                               K=c(30,6),
                               source.data = NULL){

  if (is.character(source.data)){
    all.covid.data<- read.csv(source.data)
  } else {
    all.covid.data<-source.data
  }
  select <- c("location",
              "yearweek",
              "new_cases",
              "new_deaths",
              "population"
  )

  non.countries <- c("International", "Europe", "Africa", "Asia", "European Union",
                     "Low income",  "Lower middle income", "Oceania", "South America",
                     "North America", "Upper middle income", "High income", "World")

  select.week<- all.covid.data$yearweek==week
  week.data <- all.covid.data[
    select.week
    &!all.covid.data$location%in%non.countries,
    select]

  week.data[is.na(week.data)]<-0

  require(npmlreg)
  predict.cases<- function(fit){
    population<- exp(fit$offset)
    pred<- population*exp(post(fit)$int)
    return(pred)
  }
```

```r
k1<-K[1]
k2<-K[2]

week.fit <- alldist(new_cases~1 , random=~1, offset=log(population),
                   k=k1, data=week.data,  family=poisson, tol=0.4,
                   plot.opt=0, verbose=FALSE)

predict.week.cases <- predict.cases(week.fit)
predict.week.case.rate<- predict.week.cases/week.data$population

covid.week.table <- data.frame(
  "location" =  week.data$location,
  "population" =  week.data$population,
  "cases"= week.data$new_cases,
  "fitted cases"= round(predict.week.cases,digits=digits[1]),
  "raw case rate" = round(week.data$new_cases/week.data$population,digits=digits[2]),
  "fitted case rate"= round(predict.week.case.rate, digits=digits[2])
)

if (deaths){
  deaths.fit <- alldist(new_deaths~1 , random=~1, offset=log(predict.week.cases),
                        k=k2,  data=week.data, family=poisson, tol=0.5,
                        plot.opt=0, verbose=FALSE)

  predict.week.deaths <- predict.cases(deaths.fit)
  predict.week.death.rate= predict.week.deaths/predict.week.cases

  covid.week.table <- data.frame(
    "location" =  week.data$location,
    "population" =  week.data$population,
    "cases"= week.data$new_cases,
    "fitted cases"= round(predict.week.cases,digits=digits[1]),
    "raw case rate" = round(week.data$new_cases/week.data$population,digits=digits[2]),
    "fitted case rate"= round(predict.week.case.rate, digits=digits[2]),
    "deaths"=  week.data$new_deaths,
    "fitted deaths"= round(predict.week.deaths, digits=digits[1]),
    "raw death rate"= round(week.data$new_deaths/week.data$new_cases, digits=digits[2]),
    "fitted death rate"= round(predict.week.death.rate, digits=digits[2])
  )

  return(list("covid.rates.table"=covid.week.table, "fit.cases"=week.fit, "fit.deaths"=deaths.fit))
  }

else return(list("covid.rates.table"=covid.week.table, "fit.cases"=week.fit))

}
```

Identify time span 2020-2022

```
cambodia.data <- covid.week.data[covid.week.data$location=="Cambodia",]
index<-1:159  # corresponds to 2020-2022
Dates <- cambodia.data$yearweek[index]
```

Test run algorithm for a single week

```
test<- weekly.covid.rates(Dates[1],
                          lag=0,
                          K=c(30,6),
                          deaths=TRUE,
                          digits=c(4,7),
                          source.data=covid.week.data)
```

Prepare an empty matrix to store results

```
cambodia.results <- matrix(0,159,10)
colnames(cambodia.results)<- colnames(test[[1]])
```

Computing information for Table 1

```
sum(is.na(cambodia.data$new_deaths[index]/cambodia.data$new_cases[index]))
```

```
## [1] 26
```

```
sum(cambodia.data$new_deaths[index]/cambodia.data$new_cases[index]==0,na.rm=TRUE )
```

```
## [1] 80
```

```
sum(cambodia.data$new_deaths[index]/cambodia.data$new_cases[index]> 0,na.rm=TRUE )
```

```
## [1] 53
```

Fit the model

```
for  (j in 1:159){
  rates <- weekly.covid.rates(Dates[j],
                              lag=0,
                              K=c(30,6),
                              deaths=TRUE,
                              digits=c(4,7),
                              source.data=covid.week.data)$covid.rates.table
  cambodia.results[j,]<- unlist(rates[rates$location=="Cambodia",])
  if (j%%10==0){print(j)}
}
```

```
## [1] 10
## [1] 20
## [1] 30
## [1] 40
## [1] 50
## [1] 60
## [1] 70
## [1] 80
## [1] 90
## [1] 100
## [1] 110
## [1] 120
## [1] 130
## [1] 140
## [1] 150
```

Display first 10 rows of outcome

```
cambodia.results[1:10,]
```

```
##       location   population cases fitted.cases raw.case.rate fitted.case.rate
##  [1,] "Cambodia" "16767851" "0"   "0.0063"     "0"           "0"
##  [2,] "Cambodia" "16767851" "0"   "0"          "0"           "0"
##  [3,] "Cambodia" "16767851" "0"   "0"          "0"           "0"
##  [4,] "Cambodia" "16767851" "0"   "0"          "0"           "0"
##  [5,] "Cambodia" "16767851" "1"   "0.352"      "1e-07"       "0"
##  [6,] "Cambodia" "16767851" "0"   "0"          "0"           "0"
##  [7,] "Cambodia" "16767851" "0"   "0"          "0"           "0"
##  [8,] "Cambodia" "16767851" "0"   "1e-04"      "0"           "0"
##  [9,] "Cambodia" "16767851" "0"   "1e-04"      "0"           "0"
## [10,] "Cambodia" "16767851" "1"   "0.558"      "1e-07"       "0"
##       deaths fitted.deaths raw.death.rate fitted.death.rate
##  [1,] "0"    "0"           "NaN"          "0"
##  [2,] "0"    "0"           "NaN"          "0.253776"
##  [3,] "0"    "0"           "NaN"          "0.0224833"
##  [4,] "0"    "0"           "NaN"          "0.0248292"
##  [5,] "0"    "1e-04"       "0"            "0.0001698"
##  [6,] "0"    "0"           "NaN"          "0.0080008"
##  [7,] "0"    "0"           "NaN"          "0.0169195"
##  [8,] "0"    "0"           "NaN"          "0.016475"
##  [9,] "0"    "0"           "NaN"          "0.0111814"
## [10,] "0"    "0"           "0"            "5.26e-05"
```

Produce graphical summaries

```
yul<-max(cambodia.data$new_cases[index])*1.2
plot(cambodia.data$new_cases[index], ylab="cases", xlab="week",
     main="Cambodia: Weekly case counts", type="l", ylim=c(0,yul))
segments(53,0,53,yul, col="grey")
segments(107,0,107,yul, col="grey")
```

```
text(20,.92*yul, "2020", col="grey30")
text(80,.92*yul, "2021", col="grey30")
text(140,.92*yul, "2022", col="grey30")
```

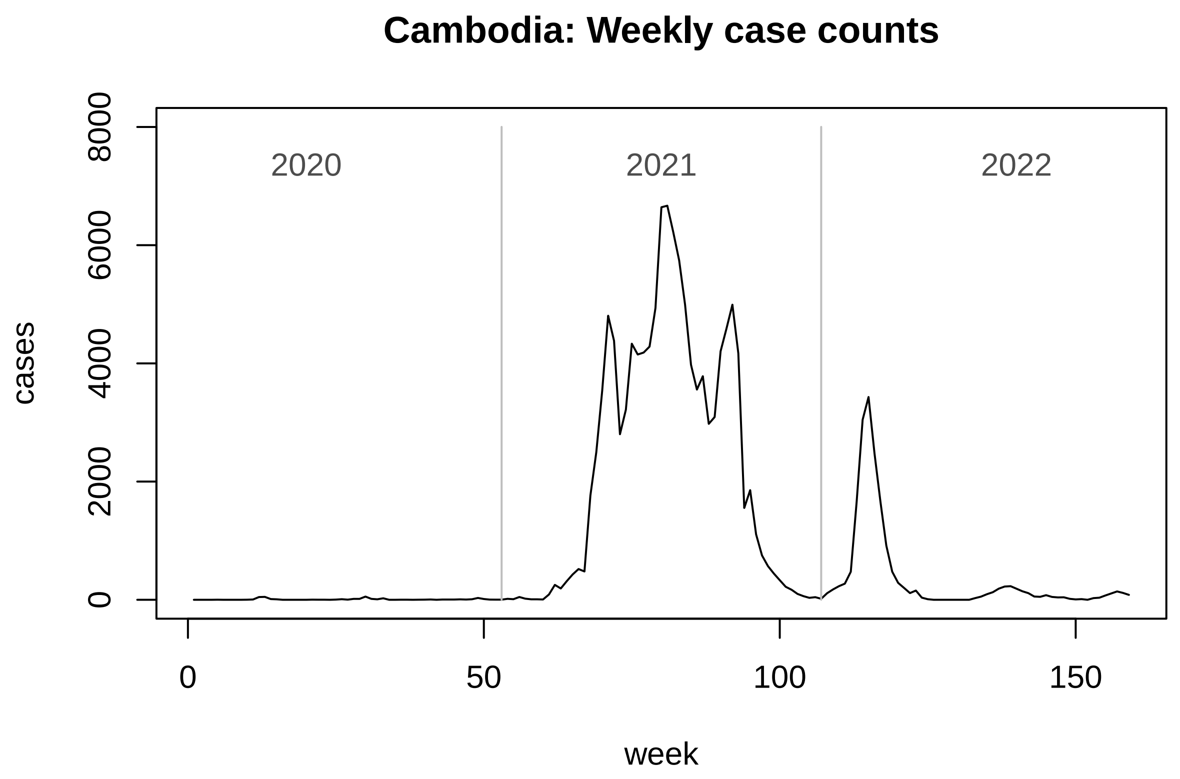


```
dul<- max(cambodia.data$new_deaths[index])*1.2
plot(cambodia.data$new_deaths[index], ylab="deaths", xlab="week",
     main="Cambodia: Weekly death counts",type="l", ylim=c(0,dul))
segments(53,0,53,dul, col="grey")
segments(107,0,107,dul, col="grey")
text(20,.92*dul, "2020", col="grey30")
text(80,.92*dul, "2021", col="grey30")
text(140,.92*dul, "2022", col="grey30")
```

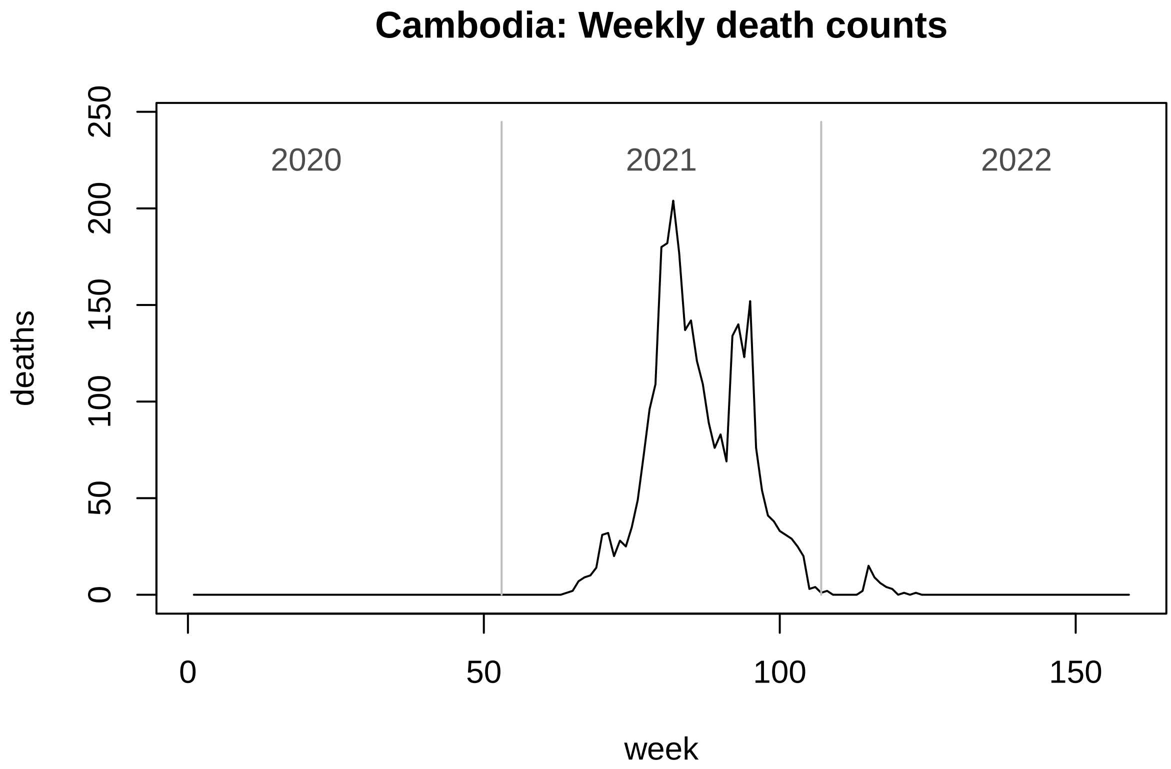


```
plot(as.numeric(cambodia.results[,"raw.death.rate"]),
     as.numeric(cambodia.results[,"fitted.death.rate"]),
     ylab="fitted death rates", xlab="raw death rates", type="p", #ylim=c(0,dful),
     col="grey30", main="Fitted vs raw weekly death rates")
abline(0,1, lwd=1.2)
```

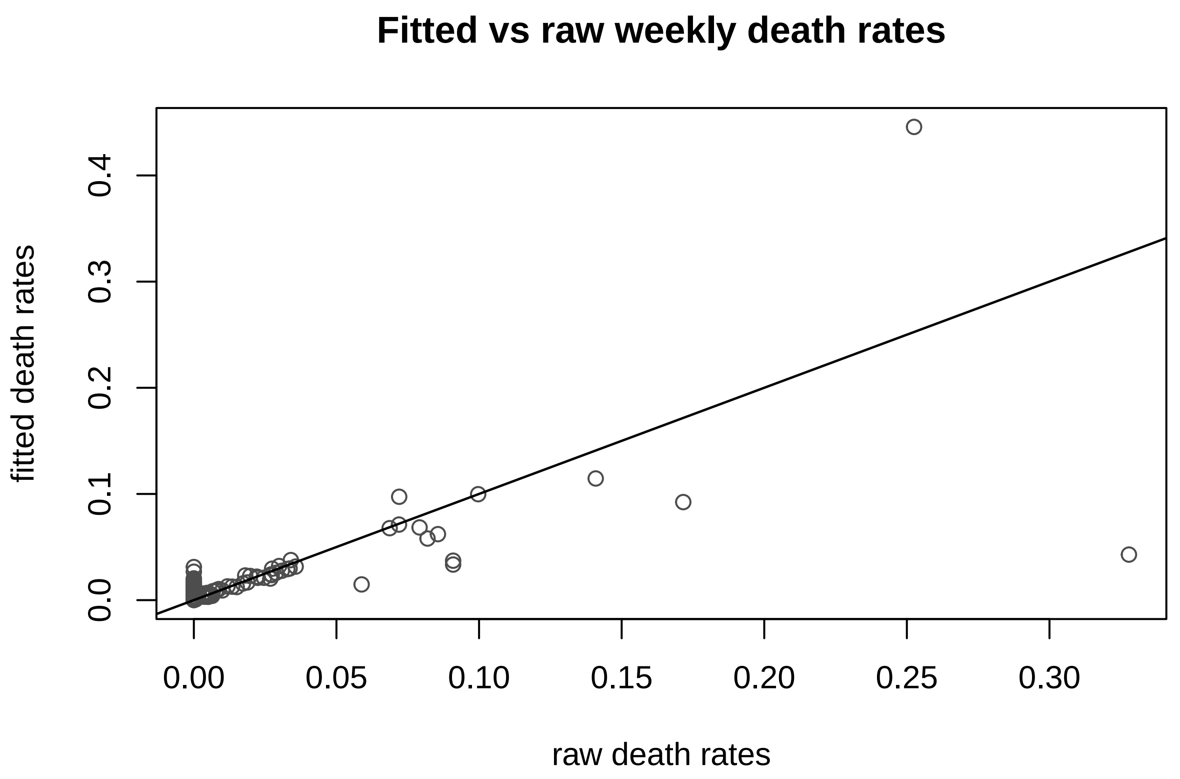


```
dful<- max(as.numeric(cambodia.results[index,"fitted.death.rate"]))*1.2
plot(as.numeric(cambodia.results[,"fitted.death.rate"]),
     ylab="fitted death rates", xlab="week", type="l", ylim=c(0,dful),
     main="Fitted weekly death rates")
segments(53,0,53, dful, col="grey")
segments(107,0,107,dful, col="grey")
text(20,.92*dful, "2020", col="grey30")
text(80,.92*dful, "2021", col="grey30")
text(140,.92*dful, "2022", col="grey30")
```

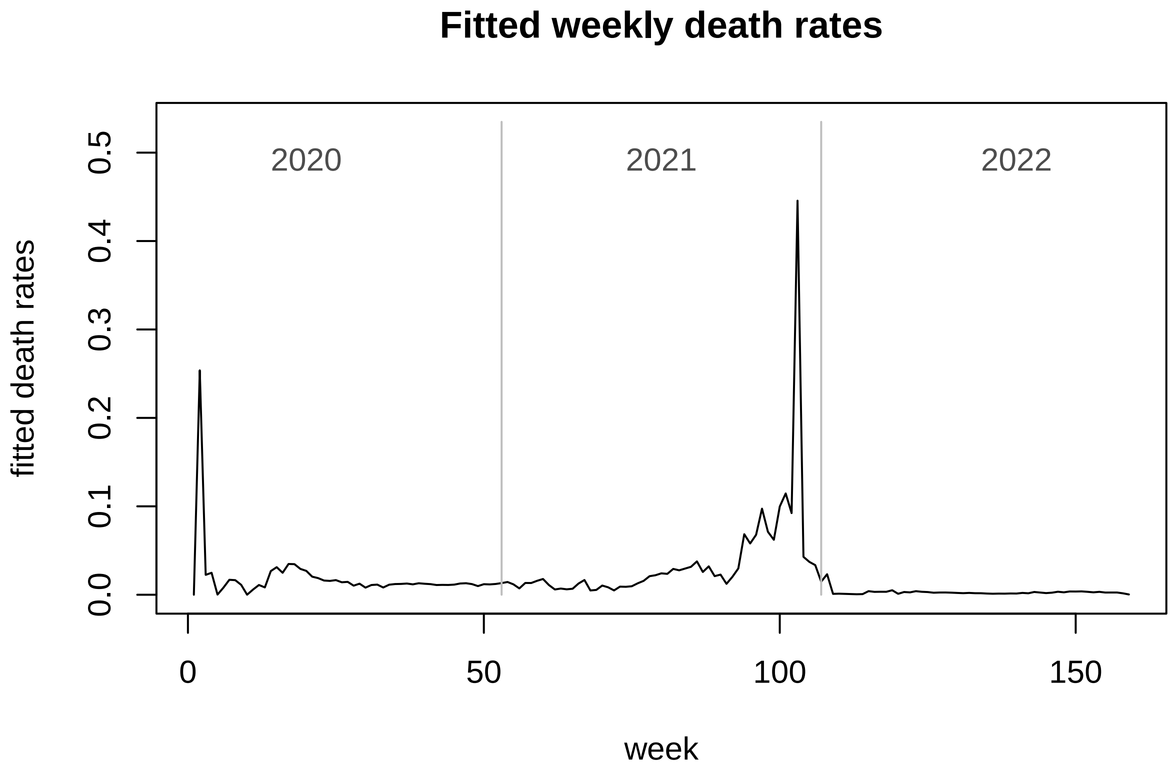
Fitted weekly death rates
2020
2021
2022
fitted death rates
0.0
0.1
0.2
0.3
0.4
0.5
0
50
100
150
week
